# A Call for a Better Understanding of Aquatic Chytrid Biology

**DOI:** 10.3389/ffunb.2021.708813

**Published:** 2021-08-04

**Authors:** Davis Laundon, Michael Cunliffe

**Affiliations:** ^1^Marine Biological Association, The Laboratory, Citadel Hill, Plymouth, United Kingdom; ^2^School of Environmental Sciences, University of East Anglia, Norwich, United Kingdom; ^3^School of Biological and Marine Sciences, University of Plymouth, Plymouth, United Kingdom

**Keywords:** chytrid, chytridiomycota, saprotroph, parasite, aquatic

## Abstract

The phylum Chytridiomycota (the “chytrids”) is an early-diverging, mostly unicellular, lineage of fungi that consists of significant aquatic saprotrophs, parasites, and pathogens, and is of evolutionary interest because its members retain biological traits considered ancestral in the fungal kingdom. While the existence of aquatic chytrids has long been known, their fundamental biology has received relatively little attention. We are beginning to establish a detailed understanding of aquatic chytrid diversity and insights into their ecological functions and prominence. However, the underpinning biology governing their aquatic ecological activities and associated core processes remain largely understudied and therefore unresolved. Many biological questions are outstanding for aquatic chytrids. What are the mechanisms that control their development and life cycle? Which core processes underpin their aquatic influence? What can their biology tell us about the evolution of fungi and the wider eukaryotic tree of life? We propose that the field of aquatic chytrid ecology could be further advanced through the improved understanding of chytrid biology, including the development of model aquatic chytrids and targeted studies using culture-independent approaches.

## Introduction

**Figure 1:**
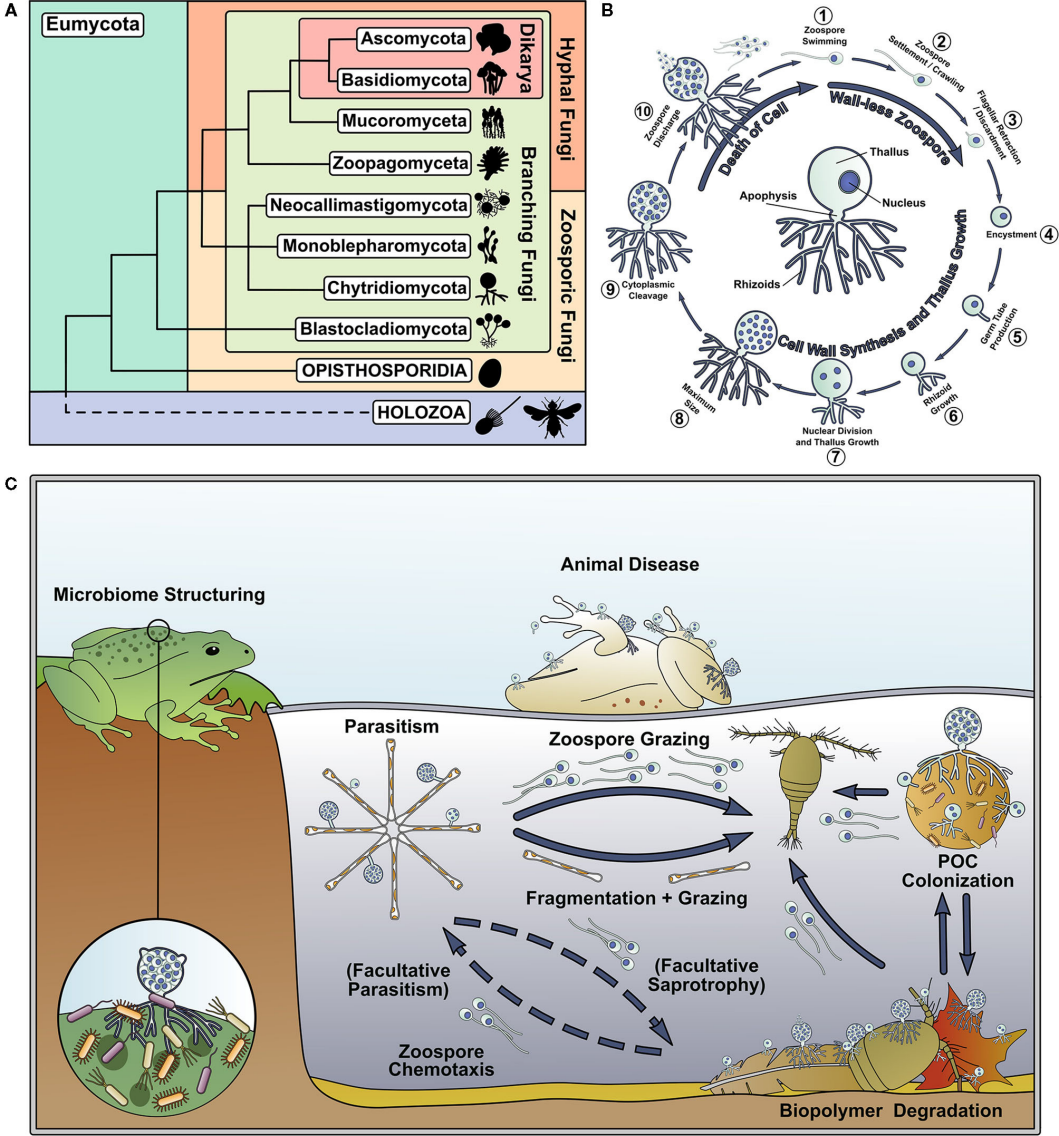
Chytrids represent an important and understudied early diverging branch of the fungal tree of life and are important components of aquatic food webs. **(A)** Phylogenetic tree of the Kingdom Fungi, adapted from the phylogeny outlined in Tedersoo et al. (2018). **(B)** Key stages of the archetypal dimorphic chytrid lifecycle. The center of the circle highlights key features of the chytrid cell anatomy. **(C)** Summary of some of the ecological roles played by chytrids in aquatic ecosystems.

The phylum Chytridiomycota (Hibbett et al., 2007) (the “chytrids”) is an early-diverging, predominantly unicellular group of fungi that use anucleate rhizoids to attach and feed on substrates, and reproduce by motile uniflagellate zoospores (Sparrow, 1960; Naranjo-Ortiz and Gabaldón, 2019) (Figures 1A,B). Chytrids are important components of aquatic ecosystems (Figure 1C), and their ecological impact has been thoroughly reviewed by previous authors (Frenken et al., 2017; Gleason et al., 2017; Grossart et al., 2019). In addition, chytrids retain biological characteristics and traits shared with their last common ancestor with hyphal fungi, making them of interest to evolutionary biologists, which has also been recently well-reviewed (Berbee et al., 2017; Nagy et al., 2017; Naranjo-Ortiz and Gabaldón, 2020). Even though aquatic chytrids have long been known to science, their fundamental biology has received relatively little attention compared to other fungi.

It is not the aim of this perspective article to review the entire fields of chytrid biology, ecology, and evolution, but to highlight recent advances related to aquatic chytrid research and knowledge gaps. It is also not within the scope of this perspective to discuss developments in chytrid taxonomy and molecular phylogeny, which have been recently covered by others (Frenken et al., 2017; Hurdeal et al., 2021). We also provide our opinion on the future direction of aquatic chytrid research, including important questions to be addressed and how this could be achieved. Our standpoint is the importance of the fundamental biology of chytrids and how increased biological knowledge could improve understanding of the ecology and evolution of aquatic chytrids.

## Biology Underpinning Aquatic Chytrid Ecology

Motile zoospores are a major feature of aquatic chytrids, which enable the targeting of trophic substrates and hosts by the propagules in a way not possible by dikaryan spores. Dissolved molecules act as chemoattractants for zoospores (Muehlstein et al., 1988; Moss et al., 2008; Scholz et al., 2017). *Batrachochytrium dendrobatidis* Longcore et al. (1999) zoospores are attracted to amphibian thyroid hormone (Thekkiniath et al., 2013) and are repelled by antifungal metabolites produced by amphibian skin bacteria (Lam et al., 2011). Zoospores have also been shown to exhibit positive phototaxis (Muehlstein et al., 1987). Cell structures, including the enigmatic chytrid rumposome, that connect the cell surface with the flagellar apparatus have been implicated in zoospore response to environmental signals (Powell, 1983), however detailed mechanisms of environmental sensing and guided motility in aquatic chytrid zoospores are currently unknown.

While the trophic range and ecological niches are established for some chytrids [e.g., *Rhizoclosmatium globosum* Petersen is a saprotroph commonly found attached to chitin-rich exuviae (Sparrow, 1960)], we know little about the degradation enzymes, mechanical processes, and physiology of nutrient assimilation. This is particularly important for degraders of recalcitrant biopolymers and hosts that are inaccessible to other heterotrophs (Kagami et al., 2014; Agha et al., 2016). Comparative genomics suggest that chytrids use a range of extracellular enzymes as part of their secretome, including carbohydrate-active enzymes (CAZymes) (Lange et al., 2019), that are yet to characterized in any biological detail.

The biochemical development of lipid-rich zoospores is important in aquatic ecosystems because of trophic transfer through the mycoloop (Kagami et al., 2007a). Zoospore lipid profiling has shown enrichment in polyunsaturated fatty acids (PUFAs) and sterols (Kagami et al., 2007b; Akinwole et al., 2014). Parasitic chytrids have PUFA profiles that are similar to their hosts, indicating direct assimilation, and new sterols that are likely synthesized *de novo* (Gerphagnon et al., 2019). These details have been instrumental in quantitative aquatic ecology, allowing the modeling of C:N:P stoichiometry and nutrient flux through ecosystems (Kagami et al., 2007b). The biochemistry of lipid anabolism and intracellular transport during zoosporogenesis, and lipid catabolism during zoospore free-swimming and encystment are largely uncharacterized.

A major knowledge gap in understanding the ecological function of aquatic chytrids from a biological view is their wider role as parasites and pathogens, particularly of algae, because most of this knowledge comes from amphibian and plant hosts. Culture-based studies have characterized the impact of *B. dendrobatidis* secretions on amphibian skin (Moss et al., 2008; Rollins-Smith et al., 2019) and investigations of growth physiology have allowed for general phenotypic profiling (Berger et al., 2005; Voyles, 2011), but quantitative biological investigations into chytrid parasites of other aquatic hosts are largely lacking.

## The Biology of Chytrids In Terms of Fungal Trait Evolution

Chytrids and their close relatives represent a key transition in the fungal kingdom from generally unicellular and rhizoidal growth toward multicellularity and hyphal growth (Berbee et al., 2017; Nagy et al., 2017). As extant chytrids exhibit ancestral characteristics of the progenitors of multicellular and hyphal fungi (Berbee et al., 2017; Nagy et al., 2017), insights into their cell biology can help infer traits associated with the origin of the archetypal fungal cell form.

Of prominence are investigations into the biology of chytrid rhizoids, as it has been hypothesized that rhizoids or rhizoid-like structures were the evolutionary precursors to hyphae (Dee et al., 2015, 2019; Kiss et al., 2019; Laundon et al., 2020). Comparative genomics has suggested that hyphae evolved in the rhizoid-bearing Chytridiomycota-Blastocladiomycota-Zoopagomycota nodes of the fungal tree (Kiss et al., 2019). Monoblepharids, a sister group to the chytrids, have aseptate coenocytic hyphal growth as their predominant cell plan (Dee et al., 2015). Cytoskeleton, cytoplasmic, and vesicular organization in the hyphae of zoosporic and dikaryan fungi suggests multiple convergent origins of hyphae from rhizoid-bearing lineages (Dee et al., 2015). In chytrids, actin polymerization and cell wall synthesis guide rhizoid morphogenesis (Dee et al., 2015; Laundon et al., 2020; Medina et al., 2020), as in hyphal growth (Gow et al., 2017; Steinberg et al., 2017; Riquelme et al., 2018). Actin cables and patches are present throughout the rhizoids of several chytrid species (Dee et al., 2019; Laundon et al., 2020; Medina et al., 2020) and inhibition of normal actin polymerization disrupts rhizoid branching causing hyperbranched paramorphs (Dee et al., 2019; Laundon et al., 2020). Inhibition of cell wall synthesis also results in similarly abnormal rhizoids (Laundon et al., 2020).

Quantitative microscopy has shown that saprotrophic aquatic chytrid rhizoids are capable of developmental plasticity and functional differentiation analogous to that characteristically displayed by mycelial dikaryans (Laundon et al., 2020). Parasitic chytrids can also have outstretched rhizoids capable of finding new algal hosts (Longcore et al., 1999) and penetrating through the frustule girdle of host diatoms (Beakes et al., 1992). These few studies into the chytrid rhizoid present a promising beginning in understanding the trophic interface of the chytrid cell for both saprotrophs and parasites, and have laid the foundation for future, quantitative cell biology in rhizoid development.

## Knowledge Gaps In Aquatic Chytrid Biology and Possible Future Directions

**Figure 2:**
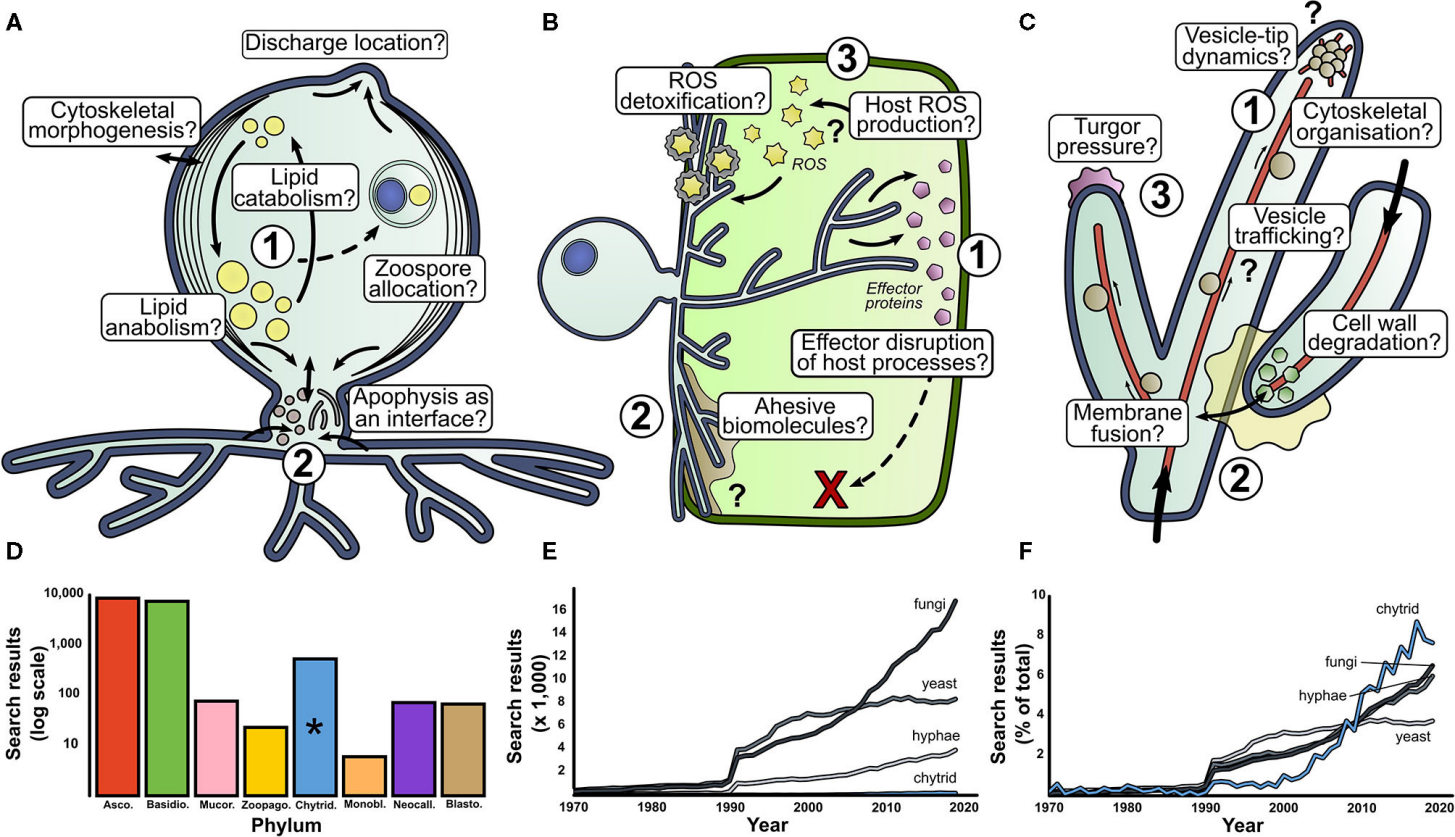
There are many fundamental questions outstanding in basic aquatic chytrid biology. Our perspective for research directions in aquatic chytrid biology concerning **(A)** cell development and lipid production, **(B)** chytrid-host interaction and **(C)** rhizoid biology. Question marks indicate biological processes whose presence and function are speculative. **(D)** Web of Science (WoS) counts for fungal phyla search terms since 1970. **(E)** WoS for search terms associated with mycology. Chytrids suffer low representation next to other mycological terms. However, when scaled as a percentage of total results, chytrids have experienced a sharp increase in attention in the past couple of decades **(F)**. *phylum Chytridiomycota.

Here we identify research questions that we think stand out as areas for investigation to help improve understanding aquatic chytrid biology, ecology, and evolution (Figure 2). This is not an attempt at an exhaustive list, but our perspective of knowledge gaps and future research directions that could stimulate interest and discussion.

*Chytrid cell biology important for aquatic ecology (* *Figure 2A* *)*

1. Lipid accumulation in chytrid zoospores is a key contributing factor to their ecological impact (Akinwole et al., 2014; Kagami et al., 2014; Gerphagnon et al., 2019). A large proportion of intracellular space is devoted to lipid storage, with evidence to suggest that lipid stores are dynamic throughout the zoospore stages of the chytrid life cycle (Powell, 1976). How are lipids synthesized and what are the anabolic pathways of lipid accumulation under different trophic conditions? How are lipid stores localized, regulated, and trafficked through the chytrid cell? What cellular processes govern the distribution and allocation of lipid reserves during zoosporogenesis? How does the balance between lipid anabolism and catabolism shift over the chytrid life cycle?
2. The apophysis is the subsporangial swelling in many chytrids that links the sporangia to the substrate attaching and feeding rhizoids (Laundon et al., 2020). Fluorescent labeling of the cell wall (Ota and Kawano, 2015) and endomembrane (Laundon, unpublished) have shown increased relative brightness in the apophysis compared to the sporangium indicating elevated activity, however the structure and function of the apophysis is largely unknown. What is the role of the apophysis in aquatic chytrids, how is the apophysis formed and what does it contain? How does the apophysis link the sporangium and rhizoids? Does the apophysis act as an interface between the reproductive and feeding structures?

*The biology behind aquatic chytrid-host interaction (* *Figure 2B* *)*

1. Genomic analysis of chytrid parasites show that they have genes that encode a suite of effector proteins and pathogenicity factors (Thekkiniath et al., 2015; Ellison et al., 2017; Farrer et al., 2017; Van de Vossenberg et al., 2019a,b). What are the functions of effector proteins and where is effector protein secretion localized? What is their phenotypic impact on host defense systems? What do chytrid parasites secrete when infecting hosts?
2. The biointerface between the chytrid and host represents the frontline of pathogenic cell biology where parasite virulence meets host defense, analogous to the biotrophic complex in other fungal parasites (Yan and Talbot, 2016). What biology characterizes the physical host-parasite interface and what is the biophysical composition of this interface from a subcellular and biostructural perspective? What proteins and carbohydrates are associated with parasite adhesion? What are the roles of turgor pressure, enzymatic degradation, and cytoskeletal organization in host penetration? How do penetrating rhizoids localize in subcellular compartments of the host, and how do they migrate through host cytoplasm? Is there division of labor in parasitic rhizoids (e.g., feeding vs. attachment) as with saprotrophic chytrids (Laundon et al., 2020)?
3. Reactive Oxygen Species (ROS) molecules are implicated in fungal pathogenicity as hypersensitive host defenses, cell signaling components, and parasite development cues (Camejo et al., 2016). What is the role of ROS in chytrid parasitic biology? Do hosts from various taxa accumulate ROS as a defense against chytrid parasitism, as earlier proposed (Canter and Jaworski, 1979)? How do chytrid parasites deal with ROS? What is the role of ROS in parasite development and virulence? Where is ROS production localized at a subcellular level?

*The rhizoid—the interface between aquatic chytrids and their substrates (* *Figure 2C* *)*

1. Our understanding of the subcellular machinery involved in hyphal development is well-characterized (Riquelme et al., 2018) and provides hypotheses to investigate similarities and differences in chytrid rhizoids (Laundon et al., 2020). Cell wall and actin proliferation have been implicated in rhizoidal development (Dee et al., 2019; Laundon et al., 2020; Medina et al., 2020), but how do these processes differ along the rhizoidal axis? To what extent do vesicle trafficking, cytoskeleton organization, and secretory machinery interact at the rhizoidal tips? Do ion gradients drive rhizoidal extension? Are dikaryan “hyphal” orthologs expressed in aquatic chytrids and how do they contribute to rhizoid morphogenesis and function?
2. Anastomosis is a rare event in chytrids and is probably associated with sexual reproduction (Miller and Dylewski, 1981). The biology of hyphal fusion is well-understood (Read et al., 2014), but what drives chytrid rhizoidal fusion? What enzymes are associated with adhesion and cell wall degradation? What intracellular processes drive membrane fusion? Are endomembrane trafficking and vesicle formation important? How are homeostasis, nutrient translocation and network resilience affected by a fused rhizoidal system?
3. In addition to a molecular understanding of hyphal development, we have a good understanding of the physical forces governing hyphal growth (Lew, 2011; Roper and Seminara, 2019) providing ground for investigations into rhizoid biophysics and fluid mechanics. Is internal turgor pressure comparable in rhizoids and, if so, how is it generated? How does cytoplasm flow through the rhizoids and what is transported with it? How elastic is the cell wall to deformation?

## Development of Model Aquatic Chytrids

Chytrids are understudied relative to dikaryan fungi, but the situation is starting to improve (Figures 2D–F). Of chytrid publications however, a majority are based on studies with the amphibian pathogenic batrachochytrids and plant pathogen *Synchytrium endobioticum* (Schilb.) Percival. This research is vital for the protection of global biodiversity and food security, however, as their roles as pathogens is unusual amongst the chytrids, it is unlikely that their biology is representative of most aquatic chytrids. We propose that other chytrids should also be developed as aquatic models.

Key model features include an available annotated genome, relatively easy laboratory culture (ideally under axenic conditions), a comparatively fast life cycle, experimental and genetic tractability, and representativeness of a major functional group (Leonelli and Ankeny, 2013; Yarden, 2016). For example, *Rhizoclosmatium globosum* (order Chytridiales) is a widespread, chitinophilic saprotroph, associated with chitin-rich particulate organic matter such as arthropod exuviae (Sparrow, 1960). It is frequently isolated from freshwater habitats (Canter, 1953) and is likely an ecologically important biopolymer degrader. *Rhizoclosmatium globosum* JEL800 was isolated by chitin baiting (Powell et al., 2019) and as an experimental organism is easy to culture, amenable to live-cell microscopy (Laundon et al., 2020), and has a rapid life cycle (~11–13 h at 22°C on rich medium) (Laundon, unpublished), making it an excellent choice to study aquatic chytrid biology. The *R. globosum* JEL800 genome is available via MycoCosm (Mondo et al., 2017), and the strain has been a model in studies on flagella retraction (Venard et al., 2020), rhizoid development (Laundon et al., 2020), and chytrid-bacteria interaction (Roberts et al., 2020).

The other major functional group of aquatic chytrids is as algal parasites (Gleason et al., 2008), some of which have been isolated into culture (Frenken et al., 2017). For example, *Rhizophydium littoreum* Amon (order Rhizophidiales) was isolated from the marine macroalga *Codium* from the East coast of the USA (Amon, 1984), is amenable to laboratory experimentation (Muehlstein et al., 1988) and has a dynamic trophic spectrum, ranging from saprotrophy to parasitism (Shields, 1990). Development of *R. littoreum* into a model chytrid could therefore shed light on the biology of marine facultative parasites. Other aquatic chytrid models could also contribute to our understanding of specific biological and ecological traits of algal parasites. For example, a stable co-cultured parasite-host system for obligate chytrid biotrophs will be necessary to fully resolve the biology of chytrid parasitism, such as the recently re-isolated *Zygorhizidium affluens* Canter (order Lobulomycetales) that infects the major spring bloom-forming freshwater diatom *Asterionella formosa* Hassall (Rad-Menéndez et al., 2018). Hopefully, sometime soon, successful cultures will also be isolated of chytrids that infect sea ice diatoms from the Arctic that have so far only been studied through culture-independent approaches and microscope observation (Hassett and Gradinger, 2016).

## Potential Limitations of Culture-Based Approaches and Complementary Alternatives to Understand Aquatic Chytrid Biology

It is likely that by studying model aquatic chytrid cultures alone we will not fully emulate the natural aquatic environment in which chytrids occur, and therefore will not develop a complete view of aquatic chytrid biology. Berbee et al. (2017) raised the point that many chytrid cultures are isolated by baiting in which a substrate [e.g., pollen, snake skin, or defatted hair (Fuller and Jaworski, 1987)] is placed in the environment for an amount of time for zoospores to attach to before the bait is retrieved and the bait-attached chytrids subsequently isolated. Depending on the bait used, it could be difficult to be certain of the natural substrates and niches of the isolated chytrids especially if maintained on complex laboratory media. Furthermore, when chytrids models are studied under axenic conditions the potential positive and negative complex interactions that take place with other components of aquatic ecosystems, such as bacteria (Roberts et al., 2020), will not be considered.

Environmental DNA (eDNA) based assessments of aquatic ecosystems have shown a vast diversity of zoosporic fungi (including chytrids) that have no cultured representatives, in some cases entire clades (Grossart et al., 2016). These cryptic chytrids that are only known from molecular surveys have been called “dark matter fungi” (Grossart et al., 2016). For example, Richards et al. (2015) surveyed marine fungal diversity across six European sites using V4 SSU sequence data and retrieved familiar operational taxonomic unit (OTU) clusters closely related to known genera including *Kappamyces* (Rhizophydiales) and *Chytridium* (Chytridiales). The molecular survey also revealed many OTU clusters from clades without cultured representatives. Some of the OTU clusters with no cultured representatives had high relative coverage in the sequence libraries suggesting that these chytrids are abundant in the samples and therefore likely important players at the various marine sites sampled. Some of the OTU clusters are part of clades that have been found in other surveys elsewhere suggesting that they are widely distributed in the marine environment. It is possible that with improved sampling effort, some chytrids only previously known via molecular surveys could be isolated into culture. A multiyear eDNA-based time-series survey of marine fungal diversity in the coastal waters off Plymouth (UK) showed that in some years a chytrid OTU (OTU 14) was prevalent only during the spring diatom bloom suggesting that the chytrid was a diatom parasite (Taylor and Cunliffe, 2016). Garvetto et al. (2019) subsequently isolated a novel species within the Rhizophydiales that infected the bloom-forming diatom *Skeletonema* and was closely related to OTU14. If we only study cultured chytrid models (as outlined above) and ignore “dark matter chytrids,” we will not be able to achieve a truly complete understanding of aquatic chytrid biology.

Alternative approaches are available to study aquatic chytrid biology that do not rely on isolated model cultures and that are able to include “dark matter chytrids.” Single-cell genomics is one option to target uncultured aquatic chytrids (Ahrendt et al., 2018). The approach is based on the isolation of a target population of cells from a complex sample, such as via fluorescence-activated cell sorting (FACS), and the subsequent extraction of DNA, genomic DNA amplification and sequencing. The approach has been used to target uncultured mycoparasitic and saprotrophic early-diverging fungi including chytrids, and because genomic-level information is retrieved, gene-based aspects of biology such as potential niche associated metabolic capability can be predicted (Ahrendt et al., 2018).

Meta-omic approaches (metagenomics, metatranscriptomics, etc.) are now widely established in aquatic microbial ecology and allow biological understanding to be established without cultivation of target organisms. Metagenomics and metatranscriptomics have been used effectively to explore the functional biology of other uncultivated aquatic fungi, including for example the potential role of marine fungi in organic matter processing (Chrismas and Cunliffe, 2020; Baltar et al., 2021). We are not aware of any studies so far that have used meta-omic approaches to specifically target and study aquatic chytrids, however this area has great potential and warrants attention.

## Concluding Remarks

Chytrids are widespread, sometimes dominant, fungi in a range of aquatic ecosystems. In addition, they are an appealing choice for evolutionary biologists understanding the position of aquatic fungi in the eukaryotic tree of life and the origins of fungal biological trait innovations. These factors have generated an interest in chytrids and a drive to understand their biology. The current increase in sequenced genomes and comparative genomics have made major contributions, but we cannot fully understand chytrid biology by genomic approaches alone. Parallel to these investigations, it is necessary to use culture-based investigations into chytrid biology and apply targeted culture-independent tools to explore aquatic chytrids *in natura*. We have outlined our perspective to bring aquatic chytrids closer to the forefront of fungal biology. This is a call not only for aquatic chytrid researchers, but also for general cell biologists to choose chytrids in their studies and take advantage of the potential of these aquatic fungi. The community should work collaboratively to achieve a comprehensive understanding of chytrid biology by combining skillsets, from taxonomists to cell biologists, and from evolutionary biologists and paleomycologists to contemporary aquatic ecologists. In these “little pots” resides great scope for discovery.

## Data Availability Statement

The original contributions presented in the study are included in the article, further inquiries can be directed to the corresponding author.

## Author Contributions

All authors listed have made a substantial, direct and intellectual contribution to the work, and approved it for publication.

## Conflict of Interest

The authors declare that the research was conducted in the absence of any commercial or financial relationships that could be construed as a potential conflict of interest.

## Publisher's Note

All claims expressed in this article are solely those of the authors and do not necessarily represent those of their affiliated organizations, or those of the publisher, the editors and the reviewers. Any product that may be evaluated in this article, or claim that may be made by its manufacturer, is not guaranteed or endorsed by the publisher.
